# Roles of Infection in Psoriasis

**DOI:** 10.3390/ijms23136955

**Published:** 2022-06-23

**Authors:** Shihui Zhou, Zhirong Yao

**Affiliations:** 1 Department of Dermatology, Xinhua Hospital, Shanghai Jiaotong University School of Medicine, Shanghai 200092, China; zhou_chihiro@sjtu.edu.cn; 2 Institute of Dermatology, Shanghai Jiaotong University School of Medicine, Shanghai 200092, China

**Keywords:** psoriasis, infection, bacterial infection, viral infection, fungal infection

## Abstract

Psoriasis is a chronic, immune-mediated disorder with cutaneous and systemic manifestations. Genetic predisposition, environmental factors, and immune dysfunction all contribute to the pathogenesis of psoriasis with host-microbe interaction governing the progression of this disease. Emerging evidence has indicated that infection is an environmental trigger for psoriasis and plays multiple roles in its maintenance as evidenced by the frequent association between guttate psoriasis onset and acute streptococcal infection. Different infectious factors act on immune cells to produce inflammatory cytokines that can induce or aggravate psoriasis. In addition to bacterial infections, viral and fungal infections have also been shown to be strongly associated with the onset or exacerbation of psoriasis. Intervention of skin microbiota to treat psoriasis has become a hot research topic. In this review, we summarize the effects of different infectious factors (bacteria, viruses, and fungi) on psoriasis, thereby providing insights into the manipulation of pathogens to allow for the identification of improved therapeutic options for the treatment of this condition.

## 1. Introduction

Psoriasis is a chronic, immune-mediated systemic disease that is genetically and environmentally associated, and it affects approximately 125 million people worldwide [1]. Typical clinical manifestations involve sharply demarcated, scaly, and erythematous plaques. Patients with moderate to severe psoriasis are at increased risk of metabolic syndrome, atherosclerotic cardiovascular disease, and depression, all of which significantly affect their quality of life [2]. Since the pathogenesis of psoriasis has not been fully elucidated, a complete cure for psoriasis is facing a great challenge.

Environmental factors, which play important roles in both the predisposition and the aggravation of psoriasis, have been revealed and appreciated. Environmental triggers of psoriasis include stress, surgery, alcohol abuse, smoking, and, most prominently, infection [3,4]. The clinical correlation between guttate psoriasis (GP) and streptococcal pharyngitis is well established, indicating a bridge between psoriasis and bacterial infection [5]. Besides bacterial infections, viral and fungal infections have also been confirmed as the key factors that may induce psoriasis [6]. In addition, changes in the skin cutaneous microbiota are one of the major features of psoriasis [7]. Recently, it has been found that gut microbiota may play an equally important role in psoriasis through the gut–skin axis [8]. Effective relief of psoriasis can be achieved through interventions for infectious factors such as antibiotics, tonsillectomy, and probiotics [9,10,11]. This not only further illustrates the important link between infection and psoriasis development, but also inspires the treatment of psoriasis.

The exact mechanism of how infection induces psoriasis remains elusive. Available studies suggest that infectious factors act on immune cells to produce inflammatory cytokines that promote psoriasis. Genome-wide association studies (GWASs) have linked multiple psoriasis susceptibility *loci* to immune-related genes, thereby providing a genetic link between psoriasis predisposition and immune deregulation [12,13,14]. Genetic cues and the successes of biologics on patients demonstrate the interleukin (IL)-23–T helper 17 (Th17) cells axis as the key drivers of psoriasis. IL-23, which transactionally activates Tyk/Jak2 and STAT3, induces the differentiation and proliferation of Th17 cells, and subsequent cytokine secretion, including IL-17, IL-21, and IL-22. These inflammatory cytokines enhance keratinocyte proliferation, stimulate angiogenic mediators and endothelial adhesion molecules, and promotes immune cell infiltration in psoriatic skin. Cross-talk between the innate and adaptive immune systems mediated by tumor necrosis factor-alpha (TNF-α) and interferon-gamma (IFN-γ) has also been implicated in the pathogenesis of psoriasis [15]. TNF-α is a proinflammatory cytokine produced by immune cells such as dendritic cells, macrophages, and T cells, thereby exerting a wide range of biological effects. IFN-γ, one of the first inflammatory mediators discovered in psoriatic plaques, is responsible for promoting antigen processing and the expression of major histocompatibility complex (MHC) class II molecules, as well as inducing the expression of proinflammatory mediators [16]. Apart from their roles in psoriasis, these cytokines have also been shown to be closely related to infection, further illustrating the role of infection as an environmental factor in triggering psoriasis. IL-17A and IL-17F protects against bacterial and fungal infections by recruiting neutrophils and inducing antimicrobial proteins at the site of infection [17,18], though IL-17C and IL-17E may vary in function in antifungal/antibacterial immunity [19,20,21]. TNF-α is central in the initial infection defense [22]. Interferons are activated in response to stimuli. For instance, acute viral infection of keratinocytes results in upregulation of type I–III IFN to impede viral replication [23,24].

Although direct evidence is still lacking, infection may be potentially associated with a high incidence of depression and anxiety in patients with psoriasis [25,26]. First, infection may elevate the incidence of psychiatric symptoms in psoriasis patients through inflammatory cytokines. Several studies have shown that pro-inflammatory cytokines in psoriasis, such as IL-6, IL-17, and TNF-α, are associated with psychiatric disorders [27,28]. Second, the microbiota–gut–brain axis can be the potential bridge between infection or microbiota dysbiosis and psychiatric or psychological problems [29]. Patients with depression and anxiety exhibit disturbances in the gut microbiota [30,31]. Moreover, depression and anxiety can be alleviated through modification in the gut microbiota [32]. However, further research is needed regarding the direct mechanisms of specific infectious factors on anxiety or depression in patients with psoriasis.

Infection that contributes to psoriasis can be divided into three main categories as follows: bacterial (*Streptococcus*, *Staphylococcus aureus*, and *Helicobacter pylori*), viral (human immunodeficiency virus (HIV), hepatitis virus, human papillomavirus (HPV), cytomegalovirus (CMV), *Zika virus* (ZIKV), and severe acute respiratory syndrome coronavirus 2 [SARS-CoV-2)), and fungal (*Malassezia* and *Candida*). Here, we review the roles of infection in the pathogenesis of psoriasis based on the three categories of infection and from two perspectives, namely, epidemiological and mechanistic. This not only provides new insights into the pathogenesis of psoriasis, but also lays the foundation for efficient diagnosis and treatment of psoriasis in the future.

## 2. Bacterial Infection

**Figure 1:**
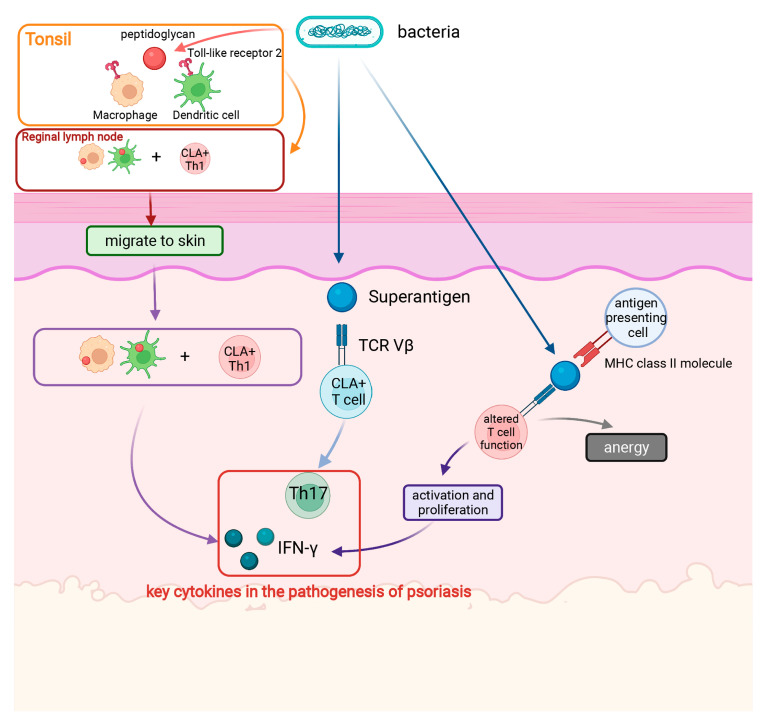
Roles of bacteria in psoriasis. Superantigens comprise a classical mechanism. Bacterial isolates generally express several superantigens. Superantigens bind to the outer surface of MHC class II proteins on the surface of antigen presenting cells, connecting them T cell receptors on the surface of T helper cells. T cells are then activated, leading to proliferation and production of cytokines (such as IFN-γ). However, overstimulation promotes T cell failure and dysfunction, which is called anergy. In addition, superantigens increase T cell expression of the skin-homing receptor, cutaneous lymphocyte antigen, which further mediates Th17-dominated responses. Another mechanism is related to peptidoglycan (PG). Bacteria in the tonsil (and/or intestine) or PG released by bacteria can be absorbed by macrophages or dendritic cells, which then migrate to the skin where PG peptides are presented to antigen-specific Th1 cell clones. Released cytokines, especially IFN-γ, act on keratinocytes. Finally, excessive proliferation and incomplete differentiation of the epidermal layer occurs. Created with BioRender.com on 24 May 2022.

The cutaneous microbiome of patients with psoriasis differs from those without psoriasis [33]. Additionally, skin samples from patients with psoriasis have reduced microbial diversity compared to healthy controls [34,35]. Gao et al. [7] reported that the most abundant and diverse phylum in psoriatic lesions is Firmicutes compared to samples from uninvolved skin of the patients and the healthy control. Conversely, the most prevalent and diverse phylum of skin samples from healthy individuals and uninvolved skin samples from patients is Actinobacteria, but it is significantly underrepresented in psoriatic lesions. Interestingly, clones representing Streptococcus are detected significantly more frequently from involved psoriatic skin compared to uninvolved skin, contrasting with the significant decrease of Propionibacterium in the involved skin. Consistently, Alekseyenko et al. [35] observed that the relative abundances of skin-associated genera are stable in healthy control samples but that they vary over time in patient samples (both lesioned and uninvolved skin areas) and are consistently different from controls; they further developed the concept of “cutaneotype” based on the relative abundance of the major phyla. Psoriasis is classified as cutaneotype 2 because the psoriasis microbiota is associated with a cutaneotype enriched with Firmicutes and Actinobacteria. In addition, there is growing evidence highlighting the importance of the gut–skin axis in the pathogenesis of psoriasis, suggesting a possible impact of the gut microbiota on psoriasis [8,36,37]. These observations suggest that a breakdown of immune tolerance due to skin dysbiosis may contribute to psoriasis (Figure 1). 

### 2.1. Streptococcal Infection

#### 2.1.1. Correlation between Streptococcal Infection and Psoriasis

*Streptococcus pyogenes* has a strong association with psoriasis [38,39,40]. Although streptococcal infection of the upper respiratory tract often promotes or aggravates psoriasis, it does not necessarily cause the condition, which indicates that there is a congenital susceptibility to streptococcal infection-associated psoriasis, especially among individuals carrying the human leukocyte antigen (HLA)-Cw6 allele [41]. 

Streptococcal infection is both an inducing factor of acute psoriasis and a stimulatory factor of chronic, persistent psoriasis. Approximately two-thirds of patients with guttate psoriasis experience a sore throat one to two weeks before disease onset with serological evidence supporting a recent streptococcal infection [42]. Additionally, the condition of patients with chronic plaque psoriasis deteriorates after tonsillitis, which is usually caused by group A *Streptococcus* [43,44]. Moreover, a subclinical tonsillar infection has also been proposed to be the immunological factor that triggers the cutaneous lesions of psoriasis and may explain their persistence. It has been suggested that the invading bacteria may enter epithelial cells and then persist there, forming a reservoir of streptococcal antigen [45,46].

#### 2.1.2. Mechanism of Streptococcal Infection Triggering Psoriasis

Aiming to identify the bridge between streptococcal tonsillitis and the T cell-mediated autoimmune response in psoriasis, Diluvio et al. [47] employed size spectratyping and sequencing of T cell receptor (TCR) β-chain variable region gene (TRBV) in streptococcal angina and skin lesions of three patients with psoriasis vulgaris. The authors compared TCR usage of psoriatic skin lesions, blood, tonsils, and tonsillar T cells fractionated according to the expression of cutaneous lymphocyte-associated antigen (CLA), and they found identical Ag-specific T cell clones in the skin and tonsils but not in the blood. The identification of repetitive TCRBV gene rearrangements further confirmed that the lesional psoriatic immune response is dominated by clonal T cell expansion, suggesting that T cells represent the link between streptococcal tonsillitis and psoriatic inflammation. These results further implied that streptococcal angina might prime and select tonsillar T cells for migration into the skin, where they are reactivated and clonally expand, thereby promoting the formation of psoriatic skin lesions.

Similar to other Gram-positive organisms, the streptococcal cell wall is predominantly composed of peptidoglycan (PG), which consists of repeating disaccharides of N-acetylglucosamine and N-acetylmuramic acid crosslinked by peptides. PG is regarded as a potentially proinflammatory cofactor in chronic inflammation [48]. There are four PG recognition proteins (PGRP-1, -2, -3, and -4), which function in antibacterial immunity. Among them, PGRP-3 and PGRP-4 are secreted by epithelial cells in the skin, tonsils, and gut. The genes encoding PGRP-3 and PGRP-4 are located on chromosome 1q21, a region containing a *locus* (PSORS4) for psoriasis [49]. Moreover, it is proposed that an antigen-specific Th1 cell response in a self-HLA-DR-restricted manner to streptococcal or other Gram-positive bacteria induce psoriasis. In this scenario, macrophages or dendritic cells, expressing receptors for PG fragments (intracellular Nod1/CARD4, intracellular Nod2/CARD15, and membrane TLR2) [50], would take up PG in the tonsils and/or gut and then migrate to the skin where they would present PG peptides to antigen-specific Th1 cells.

Another theory explaining how streptococcal infections trigger psoriasis concerns superantigens, which are extracellular protein toxins with properties that include pyrogenicity, mitogenic activity for specific T cell subsets, and the ability to increase host susceptibility to endotoxic shock and suppress immunoglobulin production. Common protein antigens activate between one millionth and one thousandth of the T cell clones in the body’s total T cell bank, while superantigens activate 2–20% of T cell clones at low concentrations and produce a strong immune response via bypassing the MHC restriction. Streptococcal superantigens include pyrogenic exotoxin and M protein [51], and the latter is the main virulence factor of group A beta-hemolytic streptococci [52]. 

Tokura et al. [53] measured the response of peripheral blood monocytes to streptococcal superantigens in vitro and found that local streptococcal infection leads to the release of superantigens and the temporary activation of related T cells. Superantigens comprise a group of microbial products that stimulate the activation and proliferation of T cells that express the Vβ fragment of the TCR and produce immune-related cytokines [54]. In the pathogenesis of *Streptococcus*-induced acute guttate psoriasis, M protein, pyrogenic exotoxin, and other superantigens have been reported to directly activate T cells through binding to the Vβ region of the TCR, leading to drip-type skin damage. However, T cell overstimulation promotes T cell exhaustion and dysfunction as well as secondary immunosuppression, which may be related to cellular immune deficiency in psoriasis [55,56]. 

Superantigen-activated T cells also produce IFN-γ, which induce keratinocytes to express intercellular adhesion molecule 1 (ICAM-1) [57]. ICAM-1 interacts with leukocyte functional antigen (LFA) to help neutrophils stay in the epidermis and form “Munro’s microabscesses”, a hallmark of psoriasis. Psoriatic keratinocytes express HLA-DR, and the interaction between ICAM-l and LFA-1 on the surface of T cells provides costimulatory signals for superantigen-activated T cells. However, different HLA allele products differ in their ability to present superantigens, leading to psoriasis in susceptible individuals after streptococcal infection, depending on the allele product.

Superantigens derived from β-hemolytic streptococci have been shown to enhance the expression of CLA in T cells, and CLA^+^ T cells express TCR Vβ2, a receptor for streptococcal superantigens. Circulating CLA^+^ T cells, a subset of memory T cells that display skin tropism, preferentially respond to antigens associated with T cell-mediated cutaneous disease. CLA^+^ T cells migrate to the skin before the psoriasis plaque forms and have been implicated in the pathogenesis of psoriasis. Using a novel ex vivo culture system, in which circulating memory CLA^+^/CLA^−^ T cells are cultured together with autologous lesional or nonlesional epidermal cells with or without streptococcal extract from patients with psoriasis or healthy controls, Ferran et al. [58] found that exposure to streptococcal extracts leads to the activation of key psoriatic T cell-derived cytokines and epidermal chemokines. These results further support the direct involvement of streptococcal infection in the pathogenesis of psoriasis. Similarly, the authors cocultured circulating memory T cells with autologous epidermal cells from patients with guttate psoriasis or healthy controls and observed a Th17-dominant response [59]. 

Finally, more than 68% of patients with psoriasis but without persistent streptococcal infection develop the chronic plaque-type of the disease, which may be related to the cross-reactive self-antigens. Keratins comprise a group of intermediate microfilaments encoded by different genes and have a similar spiral structure to and extensive homology with streptococcal M proteins. The latter was first reported approximately 30 years ago. Comparison of approximately 4200 mammalian proteins has demonstrated that human type 1 keratins show the strongest homology with the streptococcal M6 protein [60]. Similarly, although psoriasis serum recognizes skin lesion antigens by immunosorbing the Streptococcus pyogenes extract, it binds first to streptococcal antigens, and therefore, can no longer recognize its own lesion antigens [61]. The Streptococcal monoclonal antibody also reacts with skin lesions, suggesting that psoriatic skin lesions and streptococci have similar components. This further implies that molecular mimicry exists between streptococcal and epidermal components, thereby allowing T cell clones directed against streptococcal components to initiate the psoriatic process in genetically predisposed individuals. Besgen et al. [62] immunized rabbits with group A β-hemolytic streptococci, which induced the production of antibodies against several proteins expressed in keratinocytes, including ezrin, maspin, HSP27, and PRDX2. These proteins may serve as autoantigens in streptococcal infection-induced psoriasis.

Recently, Allen et al. [43] proposed that biofilms, which are loosely defined as clusters of microorganisms that adhere to biological or nonbiological surfaces, may be involved in the development of psoriasis. These researchers histopathologically analyzed tonsillectomy specimens obtained from patients with psoriasis and showed that group A streptococcal organisms persist within both intracellular and extracellular biofilms within the specimens. Furthermore, they found that in the context of psoriasis, tonsillar intracellular biofilms serve as a nidus of infection and provide antigens that stimulate skin-homing T cells through molecular mimicry. These observations help explain the relapsing and remitting nature of the psoriatic phenotype.

#### 2.1.3. Effect of Interventions for Streptococcal Infections (Antibiotics and Tonsillectomy) on the Development of Psoriasis

Antibiotics or tonsillectomy have been presented as treatments for guttate psoriasis and flare-ups of chronic plaque psoriasis. The relationship between antibiotics and psoriasis has been the subject of debate for decades with amelioration, induction, or exacerbation of psoriasis all having been reported following antibiotic therapy [63]. A significant decrease in the Psoriasis Area and Severity Index (PASI) score has been observed after long-term use of penicillin in moderate to severe plaque psoriasis, indicating the effectiveness of antibiotics in the treatment of psoriasis [9]. In contrast, although streptococci bacteria are susceptible to penicillin, the use of this antibiotic did not promote a significant improvement in guttate psoriasis in randomized controlled trials, suggesting that the effect of antibiotics on psoriasis is still uncertain [64,65].

In addition to antibiotics, tonsillectomy is applied in the treatment of psoriasis [66]. Rachakonda et al. [10] systematically reviewed 20 studies involving 545 patients from 8 countries and found that the severity of psoriasis improves in most patients after tonsillectomy. Tonsillectomized patients not only have fewer skin lesions but also need less symptomatic treatment compared to controls, indicating that tonsillectomy becomes an adjunct therapy for psoriasis [67]. However, based on the quality of the available literature, there is not enough evidence to recommend tonsillectomy uniformly for patients whose psoriasis has a possible association with tonsillitis.

### 2.2. Staphylococcus Aureus

*Staphylococcus aureus* infection aggravates psoriatic lesions in affected patients. This bacterium colonizes in psoriatic lesions in 60% of patients with psoriasis, and 60% of isolates secrete staphylococcal enterotoxins and toxic shock syndrome toxin-1 (TSST-1). Patients with toxin-positive *Staphylococcus aureus* in the skin have a higher psoriasis area and severity index score compared to those who have toxin-negative *Staphylococcus aureus* or those who lack *Staphylococcus aureus* colonization. 

Staphylococcal superantigens have been reported to play a role in the formation of psoriatic lesions [68]. Bacterial superantigens, such as staphylococcal enterotoxin-A (SEA), have been implicated in the pathogenesis of psoriasis vulgaris. MHC class II molecules are high-affinity receptors for SEA, and T cells in psoriatic skin lesions express high levels of these molecules. A case of guttate psoriasis following Kawasaki disease (KD) was reported and *Staphylococcus aureus* was isolated from the patient’s throat [69]. The case supports that superantigens derived from this bacterium are key pathogenic factors in both KD and guttate psoriasis. SEA molecules with a mutation in the MHC class II β-binding site induce protein tyrosine phosphorylation but fail to promote IFN-γ production or cytokine-mediated proliferation; in contrast, SEA molecules with a mutation in the MHC class II α-binding site induce IFN-γ production and alter the tyrosine phosphorylation profile. Both mutations abolish the cooperative effect on cytokine-mediated proliferation, suggesting that both MHC class II-binding sites are involved in the auto-presentation of SEA by psoriatic T cells. Thus, SEA directly induces the production of IFN-γ from MHC class II-positive psoriatic T cell lines [68].

### 2.3. Helicobacter Pylori

*Helicobacter pylori* has been reported to play an important role in psoriasis. Halasz et al. [70] investigated serum anti-*Helicobacter pylori* immunoglobulin G (IgG) titers in 33 patients and identified a causative relationship between this pathogen and psoriasis. In contrast, Azizzadeh et al. [71] found neither a significant relationship between anti-*Helicobacter pylori* serum IgG levels and psoriasis nor a significant difference between psoriasis severity and anti-*Helicobacter pylori* IgG levels, but rather, they reported that a significant relationship exists between the duration of psoriasis and the serum levels of IgG targeting *Helicobacter pylori*. It remains uncertain whether there is a relationship between psoriasis and *Helicobacter pylori* in both dermatology and gastroenterology. From the perspective of treatment, several case reports have indicated that psoriatic lesions clear up following the eradication of *Helicobacter pylori* infection [72,73]. Further clinical and basic studies are needed to confirm this association and the associated pathophysiological mechanisms.

### 2.4. Other Bacteria

Other organisms linked to psoriasis include the colonic bacteria *Enterococcus faecalis*, *Escherichia coli*, *Pseudomonas aeruginosa*, and *Proteus* species. However, their specific role in the pathogenesis of psoriasis remains to be investigated.

## 3. Viral Infection

**Figure 2:**
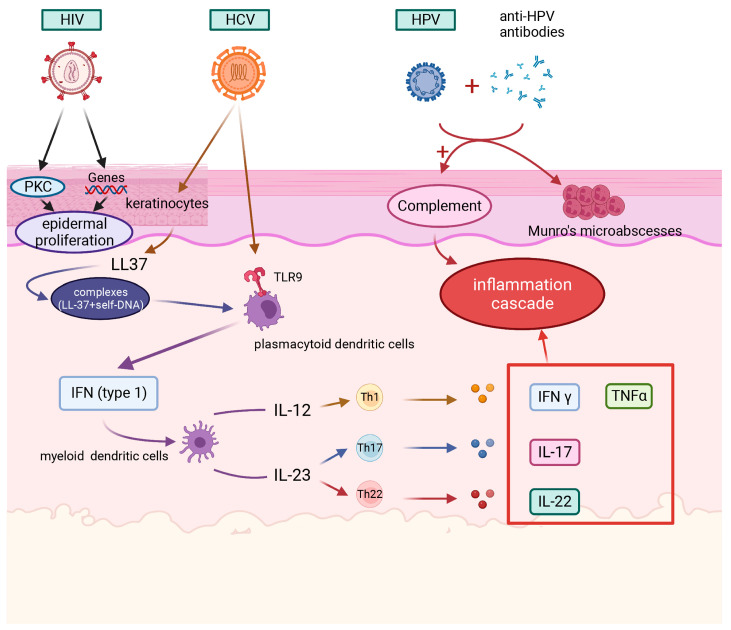
Roles of viruses (HIV, HCV, and HPV) in psoriasis. HIV: The tat gene promotes HIV replication, leading to cell proliferation through PKC-dependent or -independent pathways. HCV: HCV upregulates cathelicidin, TLR9, and IFN-γ to promote inflammation. HPV: The interaction of anti-HPV antibodies with viral proteins causes complement activation and Munro’s microabscesses. Created with BioRender.com on 21 June 2022.

Recently, Zhu et al. [6] reported for the first time the important role of virus infection in the pathophysiology of psoriasis (psoriasis vulgaris), suggesting that the dysregulation in the antiviral immune responses of hosts trigger the skin inflammatory conditions in the pathogenesis of psoriasis. Their report challenges the established view that psoriasis is caused by bacteria, especially those within the genus *Streptococcus* (Figure 2).

### 3.1. HIV

The prevalence of psoriasis in HIV-infected and acquired immunodeficiency syndrome (AIDS) patients (ranging from 1.3–5%) is higher than that in the general population, and the degree of skin lesions in these patients is also significantly greater compared with that in other patients. A cohort study involving 102,070 individuals identified HIV infection as an independent risk factor for incident psoriasis after adjusting for age, gender, and comorbidities [74]. Psoriasis in patients with AIDS is usually associated with aggravation of the disease and poor prognosis. AIDS-related psoriasis is usually difficult to treat because many treatments involve a certain degree of immunosuppression. In addition, in a case-control study, the HLA Cw*0602 allele was present in 79% of patients with HIV infection complicated by psoriasis, compared with 24.5% of HIV-positive controls. This suggests that immune dysregulation due to HIV infection is more likely to trigger psoriasis in those susceptible to the Cw*0602 allele [75].

HIV infection plays a direct role in the induction of psoriasis. The tat gene, which promotes HIV replication, has been inserted into mouse embryos, resulting in increased tat mRNA expression in the epidermis of F1 mice and hyperplasia [76]. Tat promotes cell proliferation via two distinct mechanisms. First, tat acts through a cellular pathway similar to that used by phorbol esters, such as 12-*O*-tetradecanoyl phorbol-13-acetate (TPA). Although TPA activates protein kinase C (PKC) in the presence of Ca^2+^ and phospholipids, PKC is rapidly downregulated and depleted via proteolysis. Thus, TPA has a dual effect on PKC activity in keratinocytes. However, the downstream targets have been difficult to determine. The second mechanism is independent of PKC signaling. Tat activates genes involved in controlling keratinocyte proliferation, such as *IL6*, *IL2*, *TNF*, *TGFβ1,* and superoxide dismutase (*SOD*). Activated genes change the process of programmed cell death that occur during normal epidermal differentiation, thereby promoting a proliferative state in the epidermis [77]. HIV infection also plays an indirect role in the pathogenesis of psoriasis. Dermal dendrocytes of monocytic origin that express CD4, LFA-1, and Factor XIIIa are the main HIV-infected cells in patients with psoriasis [78]. These cells produce cytokines or viral proteins, leading to epidermal proliferation and systemic immune dysfunction. On the one hand, HIV infection directly acts on the skin, while on the other hand, it causes systemic immune dysfunction, eventually leading to the occurrence of psoriasis.

Regarding the pathogenicity involved in HIV-associated psoriasis, several theories have been presented. The first theory involves cytokines [79,80,81]. Psoriasis is classically recognized as a pro-inflammatory state characterized by the overexpression of type 1 cytokines (TNF-α, IL-12, and IL-23) [80,82,83]. In addition, the levels of type 2 cytokines (IL-4, IL-6, and IL-10) are increased in HIV-infected individuals, concomitant with a reduction in the concentrations of type 1 cytokines. Thus, HIV is traditionally thought to cause a shift towards a Th2 cytokine profile. Paradoxically, IFN-γ, as a type I cytokine, is also increased in HIV-infected patients. One explanation is the Th1-Th2 shift exists only in CD4^+^ T cells. The percentage of IFN-γ-expressing cells among CD8^+^ T cells increases during HIV infection [84]. Another explanation involves the naïve and memory T cell subsets; the naïve subsets produce IL-2, and the memory subsets produce IL-4, IL-10, and IFN-γ. Therefore, the cytokine shift results from the depletion of naïve T cell subsets and the expansion of memory T cells [85].

A second theory concerns changes in the constitution of lymphocyte subpopulations. HIV infection leads to the depletion of CD4^+^ T lymphocyte numbers relative to that of CD8^+^ T lymphocytes. One study has reported that 81% of patients with psoriasis had CD4^+^ T-lymphocyte counts of less than 400. An imbalance in the CD4^+^/CD8^+^ ratio contributes to the disturbance of homeostasis, which favors psoriatic symptomatology [86]. Dysfunctional Foxp3^+^ T lymphocytes displaying a regulatory phenotype are also involved, as these cells are responsible for suppressing cellular immune responses and inducing immune tolerance.

Finally, superantigens have also been proposed to play a role in the pathogenesis of HIV infection-induced psoriasis through inducing polyclonal T lymphocyte responses along with the release of large amounts of cytokines. Superantigens bind to HLA-DR complexes and induce antigen presentation and leukocyte infiltration into skin tissue, which contributes to the cellular and chemical milieu of psoriatic plaques. Several viral proteins, namely, nef and gp120, function as superantigens during HIV infection [87,88].

### 3.2. Hepatitis B Virus (HBV)/Hepatitis C Virus (HCV)

Observed coinfection rates indicate that HBV and HCV separately trigger or exacerbate psoriasis. Some skin manifestations during HCV infection are similar to those associated with psoriasis, such as necrolytic acral erythema [89]. As early as 1995, the presence of HCV in psoriatic lesions was detected, which suggested that HCV may be a trigger for psoriasis [90]. Moreover, the prevalence of HCV infection, but not HBV infection, was greater in 12,502 psoriatic patients than in 24,287 controls [91]. The onset of psoriasis has been described in patients with chronic HCV infection after the initiation of IFN-α therapy [92].

Psoriasis is a chronic inflammatory disease mediated by the TNF-α proinflammatory cytokine [15]. TNF-α inhibitors induce a marked improvement in the skin and joint symptoms of psoriasis [93], while in HCV-infected individuals, persistent inflammation mediated by TNF-α leads to liver cirrhosis and metabolic disorders [94]. These observations indicate that psoriasis and HCV share pathophysiological factors, suggesting that the overproduction of TNF-α in HCV may trigger the onset of psoriasis. In addition, psoriatic patients with HCV are significantly less obese than those without HCV infection, yet they show a greater incidence to develop diabetes mellitus and hypertension. This phenomenon may be explained by the elevated serum TNF-α levels observed in patients with chronic hepatitis, which leads to insulin resistance and, consequently, diabetes, hypertension, and, possibly, psoriasis [95]. In addition to TNF-α, the levels of other factors, such as cathelicidin, TLR9, and IFN-γ, are increased in HCV-infected individuals, thus providing further evidence that HCV infection may predispose patients to developing psoriasis [96].

### 3.3. HPV

HPV is a small, double-stranded DNA virus found in various proliferative lesions of epithelial origin. HPV-related psoriatic lesions were first reported in 1982. Patients presented multiple common warts, flat warts, psoriatic lesions, and pityriasis-like lesions, and HPV8 was detected in these lesions [97]. A cohort study using nationwide population-based data over 12 years found a nearly 2-fold increase in incident psoriasis in patients with HPV relative to that among the general population, and the effect was significant in both sexes and patients of all age groups [98]. However, these early studies had limitations, in that the different methods utilized to detect HPV made it difficult to directly compare the data and determine the deviation due to potential confounding factors. To address these concerns, Cronin et al. [99] recruited newly diagnosed patients with psoriasis who had not previously received treatment for the condition (none had received phototherapy or immunosuppressive therapy) and further confirmed that the skin of patients with psoriasis is more prone to HPV infection than normal skin but not specifically for HPV5.

The addition of IL-17 or IFN-γ to culture medium has been shown to activate the HPV20 promoter. Based on this phenomenon, it has been hypothesized that HPV infection is latently potent in the skin instead of playing an active role in psoriasis [100]. The release of inflammatory cytokines from psoriatic lesions activates the HPV promoter, leading to active virus replication, which augments the antibody response [101].

The results of an observational study have indicated that HPV infection triggers plaque-type psoriasis and illustrated the role of nerve growth factor (NGF) in HPV-induced psoriasis [102]. It is hypothesized that HPV infection triggers an inflammatory state of the lesion, leading to upregulation of NGF. Upregulated NGF leads to pathological features of psoriasis, including keratinocyte proliferation, angiogenesis, and T cell activation [98].

Epidermodysplasia verruciformis (EV) is an inherited disease characterized by generalized flat warts or pityriasis versicolor-like macules caused by chronic infection with genus β HPV types. Patients with EV are usually infected with multiple types of HPV. EV-HPV DNA was detected in up to 90% of lesions and skin scrapings from patients with psoriasis. Psoriatic patients are considered to be a reservoir for a specific EV-HPV type, namely, HPV5 [103]. The relationship between EV-HPV and psoriasis involves [104] two genes, named *EVER1* and *EVER2*, located within the chromosomal 17qter region, which contains a dominant *locus* (*PSOR2*) for susceptibility to familial psoriasis. Patients with mutations in this region are prone both to psoriasis and EV-HPV infection [105]. Immunologically, the capsid proteins (L1 and L2) of EV-HPV act as superantigens to activate T cells [100,106]. In a previous study using an enzyme-linked immunosorbent assay (ELISA) and HPV5 L1 protein assembled into virus-like particles bearing conformational epitopes, HPV5-specific antibodies were detected in approximately 25% of patients with psoriasis (*n* = 155), which was substantially higher than the proportion in the control group. Moreover, antibodies against the L2 capsid protein of HPV5 were detected in another 5% of these patients. These results suggested that the synthesized viral peptides would be recognized by CD8^+^ lymphocytes, which could produce various proinflammatory cytokines and promote inflammation. Alternatively, the interaction between anti-HPV5 antibodies and EV-HPV proteins leads to complement activation and the accumulation of polymorphonuclear leukocytes in the stratum corneum and the formation of Munro’s microabscesses [106]. Two other proteins of EV-HPVs, namely, E6 and E7, play a role in keratinocyte proliferation. Antibodies against these two oncoproteins can be detected by radioimmunoprecipitation assay using in vitro-translated proteins [105]. Additionally, EV-HPV induces or aggravates psoriasis in cooperation with other microorganisms, such as streptococcal bacteria. The thickened epidermis, especially the stratum corneum, provides a suitable environment for HPV.

As an increasing number of people are being vaccinated with the nine-valent HPV vaccine, it will be interesting to see whether this will change the prevalence of psoriasis, especially among young women.

### 3.4. CMV

CMV is a widespread infectious agent that affects approximately 50% of the human population and is one of the most immunodominant antigens encountered by the human immune system [107]. CMV is latent in all parts of the body and is activated when the body’s immune function is low. Once activated, CMV inhibits cellular immunity. CMV activation is not only the result of active psoriasis but also an aggravating factor of the condition [108]. The main manifestations include a reduction in the number of Th cells in peripheral blood, an increase in the number of suppressive T (Ts) cells, and a reversal of the Th/Ts ratio, which further aggravates immune dysfunction. Accordingly, CMV-specific T cells are found in a considerable number in peripheral blood, often in the order of 1–2% of the total CD4^+^ and CD8^+^ T cell repertoire. CMV induces antiviral immune responses, and CMV-infected cells directly produce cytokines, such as TNF-α, or increase leukocyte activation and cytokine secretion by upregulating the expression of adhesion molecules, thereby promoting inflammation [109]. CMV superantigens have been identified, and superantigen chemotaxis represents an important factor in the development of acute psoriasis [110].

### 3.5. ZIKV

ZIKV is an emerging arthropod-borne virus in tropical and subtropical areas worldwide. ZIKV directly triggers the onset of generalized pustular psoriasis by stimulating keratinocyte-derived mediators of inflammation and a poly-functional T cell-driven immune reaction in the cutaneous milieu [111]. However, it is still uncertain whether there is a clinical relationship between ZIKV infection and psoriasis; thus, the observation may be a coincidence as there is currently no known pathological pathway common to both ZIKV infection and psoriasis that can explain a relationship between them [112].

### 3.6. Severe Acute Respiratory Syndrome Coronavirus 2 (SARS-CoV-2)

At the end of 2019, a novel coronavirus designated as SARS-CoV-2 caused an outbreak of coronavirus disease 2019 (COVID-19). Gananandan et al. [113] reported the first case of an acute guttate flare of chronic psoriasis secondary to confirmed COVID-19 infection. Moreover, several studies have reported new-onset psoriasis in the context of COVID-19 infection [114,115]. The possible mechanism of COVID-19 infection leading to psoriasis flare-ups is stimulation of toll-like receptor 3 by viral RNA, leading to dysregulation of the innate immune response and production of IL-36-γ and CXCL8 [116,117,118]. In addition, psoriasis may be exacerbated due to the hyperinflammatory state of COVID-19 patients [119].

## 4. Fungal Infection

Some clinical phenomena have suggested that a relationship may exist between psoriasis and fungi. For example, specific *Malassezia* species have been associated with particular subtypes of psoriasis, such as *Malassezia* yeasts with guttate and scalp psoriasis [120,121]. In addition, infant diaper psoriasis has been linked to *Candida albicans* infection [122]. These observations led Rosenberg et al. [123] to propose that fungi, such as *Malassezia ovalis*, which is regularly present in the scalp, activate the alternative complement pathway in psoriasis. Accordingly, the authors treated four cases of scalp psoriasis with ketoconazole and found that, after four weeks, the lesions had improved and no fungus was found in the scales [124]. Numerous epidemiological studies have demonstrated that the relationship between psoriasis and fungal infection mainly involves *Malassezia* spp. and *Candida albicans*, and most of the sites of fungal infection in psoriasis patients are sebum overflow sites and folds, which are related to the characteristics of the above two fungi [125,126].

### 4.1. Malassezia

*Malassezia*, a fungal genus that currently comprises 18 species and numerous functionally distinct strains, are lipid-dependent basidiomycetous yeasts and integral components of the skin microbiome [126]. Members of this genus are resident on the surface of the human body and can easily colonize the chest, back, scalp, and other parts that have an abundance of sebaceous glands [126]. As early as the 19th century, it was proposed that *Malassezia* spp. may be involved in the pathogenesis of psoriasis. The presence of loose scales and a rich lipid composition, combined with long-term glucocorticoid application, make psoriatic skin lesions a good site for the growth and reproduction of *Malassezia* yeasts. The severity of psoriasis has been reported to be greater among patients in whom *Malassezia* was isolated from skin lesions than in those in whom *Malassezia* was not identified [121,127].

Several studies have shown that the application of a suspension of heat-inactivated *Malassezia ovalis* causes erythema and white scales to appear on the dorsal skin of rabbits. Microscopically, the pathological changes were similar to those typically observed in psoriasis, such as parakeratosis, dilation of capillary vessels in the upper papillary dermis, changes in the numbers of polymorphonuclear leukocytes around the dermal vessels, and discrete collections of polymorphonuclear leukocytes clumped in the parakeratotic scale [123]. Furthermore, similar to that observed with the Kobner phenomenon, cell fragments of *Malassezia* spp. topically applied to the skin of psoriasis patients induce new psoriatic plaques [128]. Because polymorphonuclear leukocyte infiltration in the dermis is the initial manifestation of psoriasis, Bunse et al. [129] used *Malassezia* as a chemical inducer and observed its chemotactic effect on polymorphonuclear leukocytes; they found that *Malassezia* exerts a significantly greater chemotactic effect in patients with psoriasis compared to that in the control group, with the effect being specific to *Malassezia*.

Some researchers have considered psoriasis to be an autoimmune disease characterized by a Th1/Th2 cytokine imbalance [120]. Elevated expression of proinflammatory Th1 cytokines and low expression of Th2 cytokines play a role in the onset, maintenance, and recurrence of psoriasis. *Malassezia* can induce the occurrence and deterioration of psoriasis by affecting the differentiation of Th cell subsets in patients with this condition. The hydrophobic components of *Malassezia* stimulate the expression of proinflammatory factors in human epidermal keratinocytes, resulting in Th1-biased differentiation of Th cell subsets in peripheral blood. Using human keratinocytes and skin biopsies from *Malassezia furfur*-positive and -negative psoriasis-affected patients, Baroni et al. [130] found that this fungus upregulates the expression of transforming grow factor-β1 (TGF-β1), integrin chain, and HSP70 in human keratinocytes, subsequently affecting cell migration and proliferation.

### 4.2. Candida

*Candida* species are constituents of the normal human microbiota and colonize both the skin and mucosal membranes of the gastrointestinal and genitourinary tracts [131]. To date, over 200 species of *Candida* have been identified, among which *C albicans*, *Candida glabrata*, *Candida tropicalis*, and *Candida parapsilosis* are responsible for most candidal infections. *Candida albicans* has been reported to be associated with psoriasis [132]. For instance, there is a higher rate of concomitant fungal infections in patients with nail psoriasis, and enhanced *Candida* colonization is associated with more severe disease states [133].

The surface proteins of *Candida albicans* act as superantigens, leading to the activation of T lymphocytes with specific Vβ families independently of antigen presentation as well as the excessive release of proinflammatory cytokines [132]. Among these cytokines, IL-23 promotes the proliferation and survival of Th17 cells, which are involved in the defense against *Candida albicans*. In turn, Th17 cells release IL-17, which recruits neutrophils and helps to clear *Candida* spp. through the release of large amounts of antimicrobial peptides, direct phagocytosis, and the formation of neutrophil extracellular traps, indicating that IL-17 plays an important role in immunological protection against infections, especially those associated with *Candida* spp. Although several monoclonal antibodies targeting IL-17A or its receptors have been used to treat psoriasis [134], the incidence of *Candida* infections in patients treated with these antibodies is increased.

## 5. Discussion and Limitations

Psoriasis is a common skin disorder that brings a physical and psychological burden to affected patients. Psoriasis is commonly associated with symptoms, such as itching or burning sensations, and the disease burden is further increased by several comorbidities, including metabolic syndrome and cardiovascular disease. For patients whose psoriatic lesions occur in areas of functionally crucial areas, such as the face, palms, soles, and genitalia, the disease often has a tremendous negative psychological impact on the patient and even brings about suicidal tendencies. These observations stress the importance of fully understanding the pathogenesis of psoriasis to identify appropriate treatment options. Because the expression of psoriasis is dependent on gene-environment interactions, it is crucial to study the mechanisms by which environmental triggers act on psoriasis. Common environmental triggers include stress, infections, stress, smoking, alcohol consumption, and medications. Among them, infection is an important environmental trigger. This review has focused on discussing the role of infection in the pathogenesis of psoriasis. Although the traditional view holds that bacterial infection plays a major role in this process, recently, viral and fungal infections have increasingly been found to associate with the pathophysiology of psoriasis. The theory of superantigen involvement is associated with both bacterial and viral infection. However, current studies investigating psoriasis from an infection perspective are still relatively limited in terms of the microorganisms involved and are mostly limited to epidemiological studies rather than an in-depth exploration of the mechanisms. Therefore, more research is needed in the future to focus on the direct relationship between infectious factors and psoriasis. In the treatment of psoriasis, nanotechnology enhances the efficiency of local drug delivery and limits the systemic use of immunosuppressive drugs by improving drug penetration [4,29]. If nanotechnology can be used to deliver infection-controlling drugs, it could be a viable and efficient way to treat psoriasis in the future. To some extent, if the role of infection in psoriasis is better understood, routine testing for infectious factors in the diagnosis of psoriasis can be increased and treatment can be better targeted.

## Data Availability

Not applicable.
